# Psychological First Aid in Schools to prepare staff for supporting student mental health in disasters and critical incidents: protocol for a cluster-randomised controlled trial

**DOI:** 10.1136/bmjopen-2026-121438

**Published:** 2026-11-11

**Authors:** Emily Macleod, Sonia Curll, Philip J Batterham, Tegan Cruwys, Lou Farrer, Melinda Goodyear, Timothy Heffernan, Pauline Kostelas, Bradley Morgan, Alyssa Morse, Sonia McCallum, Dave S Pasalich, Marg Rogers, Rachael Rodney, Bree Turley, Alison L Calear

**Affiliations:** 1 Centre for Mental Health Research, National Centre for Epidemiology and Public Health, College of Law, Governance and Policy, Australian National University, Canberra, Australian Capital Territory, Australia; 2 School of Medicine and Psychology, Australian National University, Canberra, Australian Capital Territory, Australia; 3 Emerging Minds, Adelaide, South Australia, Australia; 4 School of Archaeology and Anthropology, ANU, Canberra, Australian Capital Territory, Australia; 5 School of Built Environment, UNSW Sydney, Sydney, New South Wales, Australia; 6 Department of Education, New South Wales, Sydney, New South Wales, Australia; 7 School of Education, University of New England, Armidale, New South Wales, Australia; 8 Manna Institute, Armidale, New South Wales, Australia; 9 Fenner School of Environment & Society, Australian National University, Canberra, Australian Capital Terriorty, Australia

**Keywords:** MENTAL HEALTH, Child, Adolescent, Schools, Stress, Psychological

## Abstract

**Introduction:**

Disasters can disrupt childhood development and increase long-term mental health risks. Although most children and adolescents recover with appropriate support, evidence for effective interventions to protect youth mental health in disasters remains limited. This study aims to evaluate the effectiveness of Psychological First Aid in Schools (PFA-S), a school-based training programme designed to prepare primary and secondary school staff to support student well-being during and after disasters and critical incidents.

**Methods and analysis:**

A multi-informant, mixed-methods design will be used, including a two-arm cluster-randomised controlled trial comparing PFA-S to a wait-list control condition. Eighteen public primary and secondary schools in urban and regional Australia will be randomly assigned to either the intervention (n=9) or wait-list control (n=9). All teaching, well-being and support staff at participating schools (estimated n=360; 20 per school) and all students aged ≥5 years with parent/carer consent (estimated n=1260; 70 per school) will be eligible to participate. The intervention will involve 2 hours of PFA-S staff training. Survey assessments will occur at baseline, postintervention (1 month after baseline) and follow-up (4 months after baseline). Participating staff and students aged ≥8 years will complete these surveys. The primary outcome will be staff preparedness to support student well-being in disasters (PFA-S knowledge, attitudes and skills). The secondary outcome will be general disaster preparedness. Exploratory outcomes will include actual staff use of PFA-S and student perceptions of PFA-S support. Predictors and moderators of intervention effects will be examined. Implementation data will be collected from staff via post-training surveys and semistructured interviews at follow-up, and from students aged ≥5 years via follow-up focus groups. Mixed-model repeated measures analyses will compare changes in staff preparedness and other outcomes between groups, accounting for clustering within schools. If a disaster or critical incident occurs during the study, data collection procedures will be adapted to explore real-world application of PFA-S.

**Ethics and dissemination:**

Ethics approval was obtained from the Australian National University Human Research Ethics Committee (protocol 2025/0018) and the New South Wales Department of Education State Education Research and Partnerships (SERAP 3544) overseeing participating schools. Informed consent will be obtained from all participants and their guardians. Results will be disseminated through academic publications, conferences, a summary report for schools and to the public through a project website and social media.

**Trial registration number:**

ACTRN12626000343303.

**STRENGTHS AND LIMITATIONS OF THIS STUDY**

- The cluster-randomised controlled trial design conducted in real-world school settings strengthens external validity and generalisability to diverse educational contexts.
- The mixed-methods approach evaluates both effectiveness and implementation outcomes, enhancing practical relevance for school-based delivery.
- Multiple stakeholder perspectives (school staff, students, facilitators and leaders) enable holistic assessment of acceptability, feasibility and impact.
- Children and adolescents are included as both advisors and participants, directly incorporating student voices often underrepresented in disaster intervention research.
- Potential selection bias in school, staff and student participation may limit representativeness across geographic or socioeconomic contexts.

## Introduction

 During childhood and adolescence (referring here to ages 5–18, also often described as ‘youth’), disasters pose significant threats to physical, social, emotional and educational development. Disasters are events or series of events with consequences that overwhelm the capacity of the community to cope and give rise to secondary stressors and adversities.^1 2^ In this paper, the term ‘disasters’ is used inclusively to encompass climate-related events, such as extreme weather events and earthquakes, and other critical incidents, including transport accidents, acts of violence and sudden deaths affecting a community. Children and adolescents exposed to disasters often experience academic delays^3 4^ and report higher rates of depression and anxiety compared with adults.^5^ Disaster exposure increases the risk of mental disorders that can persist into adulthood,^6^ creating long-term health system burdens and substantial individual, economic and societal costs.^7^ Disasters often disrupt protective factors, such as educator support, health services and community programmes, intensifying their impact on children and adolescents.^8^

Australia’s youth mental health services are already at or beyond capacity, managing complex and often severe needs.^9 10^ Recent events, including the 2019/2020 bushfires, COVID-19 pandemic and 2022 eastern floods, have exacerbated pressures on the mental health system.^9 11^ With climate change driving more frequent and severe disasters for the foreseeable future, these capacity constraints will only worsen, making effective disaster mental health prevention strategies critical to protect youth mental health and well-being.

Most children and adolescents can recover from disasters with appropriate support.^12^ However, there is limited research on effective tools to facilitate youth recovery.^13^ Existing disaster preparedness programmes rarely incorporate mental health components,^14^ and youth voices and needs remain underrepresented in disaster policy and research.^15 16^ Children and adolescents often lack agency in our society, especially during disasters,^17^ relying on system supports such as parents, educational institutions and community services.^18^ However, disasters frequently disrupt the very support systems that young people depend on most.^19^

Schools are uniquely well-placed to support young people’s mental health during disasters. They are accessible, provide continuity and trusted relationships, and play a central role in students’ routines and communities.^20^ However, school staff frequently report curriculum and time constraints and feeling inadequately prepared to address disaster-related distress in students.^10^ With the right training, schools could deliver early mental health support to students during disasters and facilitate timely access to community support,^21^ ultimately reducing the demand for more intensive psychological interventions delivered by specialists.

Psychological First Aid (PFA) offers a promising framework for providing school-based, preventive mental health support in disasters. PFA is an evidence-informed, universal early intervention designed for delivery by non-specialist frontline workers, such as school staff, during and immediately after potentially traumatic events.^22^ Developed by disaster mental health experts, PFA forms a key component of emergency management protocols and is grounded in research on risk and resilience after trauma.^22 23^

Building on international best-practice PFA principles, in a collaboration between researchers and the New South Wales (NSW) Department of Education (DoE), we have developed an Australian-specific Psychological First Aid in Schools (PFA-S) programme. PFA-S focuses on preparedness, equipping school staff with skills to reduce student distress, promote adaptive coping, identify mental health needs and facilitate access to community supports, while also supporting staff to manage their own well-being in disasters. The ultimate goal is prevention: reducing child and adolescent distress and mental health difficulties. PFA-S is designed to be flexible and adaptable across developmental stages and cultural and community contexts, including rural and remote areas disproportionately affected by climate disasters and socioeconomic disadvantage.^24^ Early access to mental health support aligns with disaster resilience frameworks^20^ and can improve treatment outcomes while reducing social, emotional and academic difficulties relating to untreated mental disorders.^25^ Although PFA-S was designed primarily to enable staff to support students after climate-related disasters, it also applies to critical incidents such as transport accidents, acts of violence and sudden deaths.^26^

While PFA-S may seem a commonsense approach, history has taught us that any mental health intervention must be carefully designed, implemented and evaluated to prevent harm. For example, psychological debriefing was once assumed to prevent post-traumatic stress disorder (PTSD) after disasters, but a Cochrane review found that it did not improve PTSD symptoms^27^ and may actually increase the risk of PTSD under certain circumstances.^28^ For children and adolescents, there is not enough empirical evidence to demonstrate either benefit or harm of psychological debriefing,^29^ but there is evidence that some general school-based universal mental health interventions can have potential negative effects.^30 31^

Despite widespread adoption of PFA as a frontline disaster mental health intervention, there is a concerning lack of evidence for its effectiveness, particularly for student populations and school settings.^32^ A US pilot study found school nurse-delivered PFA reduced depression symptoms after a flood, though PTSD symptoms remained unchanged.^33^ Another study reported reductions in youth post-traumatic stress (parent-reported) following PFA delivered to displaced youth after Hurricane Katrina.^34^ From a provider perspective, online PFA training improved school counsellors’ knowledge, perceived competence and preparedness to provide psychological support in future disasters.^35^ While encouraging, these studies rely on non-experimental designs, limiting conclusions about efficacy. Rigorous controlled trials are urgently needed to establish the effectiveness of PFA interventions for school-aged children and the professionals who support them in disasters.

This research will generate the first robust evidence for the effectiveness of PFA-S in schools through a cluster-randomised controlled trial (RCT) design. Our study will evaluate staff preparedness to support student well-being in disasters and prevent ongoing psychological distress, focusing on PFA-S knowledge, skills and attitudes and psychological preparedness for disasters. We will also identify moderators and predictors of intervention effects, factors influencing implementation and areas for programme improvement across individual, school and disaster contexts.

An innovative and central feature of this study will be the inclusion of child and adolescent perspectives on receiving PFA-S style support. As children develop, they gain the capacity and skills to meaningfully participate in disaster preparedness, response and recovery efforts,^36^ representing an untapped resource for strengthening disaster management capabilities.^16^ Empowering youth in disaster contexts not only promotes their agency and resilience but also enhances the effectiveness and relevance of support strategies.^37^ While PFA provides a framework for adults to foster youth agency in disasters, no research has explored how youth themselves perceive this support. This study will generate world-first data from child and adolescent end-users, offering critical insights into how effectively PFA-S meets their disaster mental health needs and where gaps remain.

### Aims and hypotheses

The primary aim of the trial is to: (1) evaluate the effectiveness of PFA-S training in preparing school staff with the knowledge, skills and attitudes needed to support students’ psychological well-being in the context of disasters. Based on prior research,^32 38–40^ it is hypothesised that staff who receive PFA-S training will report higher levels of PFA-S preparedness (primary outcome) and general psychological preparedness for disasters (secondary outcome) at postintervention and 4 month follow-up, compared with those in a wait-list control condition.

Exploratory aims are to: (2) identify predictors and moderators of intervention effects including prior disaster experience, individual factors (self-efficacy, mental health, mental health literacy) and school factors (school identification, relationships)—prior research has identified these factors as relevant to psychological preparedness, disaster mental health and intervention success^11 39 41–44^; (3) examine implementation outcomes, including fidelity, feasibility, acceptability, barriers and facilitators to implementation and opportunities for programme improvement and (4) investigate actual use of PFA-S to: (a) manage general student distress and (b) manage student distress in the context of disaster if one occurs during the study period.

A final yet key aim of this trial is to: (5) explore child and adolescent perspectives on school-based mental health support in disaster contexts, including their experiences of receiving PFA-S supports, associated mental health outcomes and additional needs for disaster preparedness and well-being.

## Methods and analysis

This study protocol complies with the Standard Protocol Items: Recommendations for Interventional Trials (SPIRIT) guidelines (see online supplemental material).

### Study design

A two-arm cluster-RCT using a type 1 hybrid implementation-effectiveness design^45^ and a superiority framework will be conducted across 18 public primary and secondary schools, with schools randomly allocated in a 1:1 ratio to either the intervention group (n=9) or a wait-list control group (n=9). Effectiveness will be evaluated at three time-points: baseline, post-intervention (1 month after baseline) and follow-up (4 months after baseline). The waitlist framework is an ethical approach that guarantees staff access to the intervention, provides more data (larger N) regarding implementation outcomes, while also incentivising participation and minimising attrition.

An implementation evaluation will be conducted alongside the efficacy trial to assess feasibility and acceptability, barriers and facilitators to implementation, fidelity and opportunities for programme improvement. Data will be collected via post-training evaluation surveys for staff, facilitators and school leaders (eg, principals), as well as student focus groups and staff semistructured interviews conducted after the 4-month follow-up.

### Participants and recruitment

We will recruit 10 primary and 8 secondary schools from the state of NSW in Australia via the intervention provider, the NSW DoE. Recruitment will intentionally oversample regional, rural and remote school communities to ensure representation of areas at increased risk of experiencing climate-related disasters and reduced service access. All teaching, well-being and support staff at participating schools will be invited to complete surveys (estimated n=360; 20 per school). All students aged ≥8 years will be eligible to assent to survey participation, pending parent/carer consent (estimated n=1260; 70 per school). Sample size estimates are based on prior analogous trials conducted by the research team, which achieved recruitment rates of 30%–50%.^46 47^ Where needed, pragmatic selective recruitment (eg, selected grades) may be used to align with school capacity to support research activities. To mitigate risk of completion bias (ie, control group being less likely to complete surveys than intervention groups), we will use strategies to support research uptake, including advisory group consultation, collaboration with school stakeholders and departmental recognition for professional development. Participants at each randomised school who do not complete baseline assessment remain eligible to enrol at subsequent assessment timepoints.

Recruitment for qualitative components will include purposive sampling of staff from intervention schools for semistructured interviews (estimated n=20, 2–3 staff per school) and students ≥5 years for focus groups (estimated n=45, 5 students per school) at 4-month follow-up. Sampling will aim to capture diverse perspectives across primary and secondary schools, staff roles, student grades and school community contexts (eg, remoteness, disaster risk). Potential staff interviewees will include well-being staff, principals and other executives and classroom teachers. Potential student focus group participants will be selected in consultation with school staff to include diversity and maximise group dynamics. Priority will be given to students from intervention schools and those attending schools selected for diversity in remoteness (Australian Bureau of Statistics Remoteness Structure),^48^ disaster risk (Australian Disaster Resilience Index),^49^ school decile,^50^ gender and cultural background.

### Power and sample size

Power and sample size calculations are based on the primary outcome of PFA-S preparedness. Based on a recent review of school-based mental health training programmes,^40^ we anticipate an effect size of d=0.57 for knowledge and d=0.36 for attitudes. To detect these effects with 80% power at alpha=0.05, a minimum staff sample of n=164 is required, prior to adjusting for attrition and clustering. For the secondary outcome, general psychological preparedness for disaster, there are no prior analogous school trials predicting effect sizes. Given some conceptual overlap between the primary and secondary outcome, we anticipate similar effect sizes for both primary and secondary outcomes. Assuming typical session sizes of n=20 staff per school, recruiting 18 schools (9 intervention/9 control; N=360) will provide sufficient power, accounting for up to 20% attrition^51^ and clustering with an intraclass correlation coefficient of 0.03. Attrition is expected to be low due to high levels of departmental buy-in and supported access to the convenience sample. Power calculations were not conducted for exploratory outcomes.^52 53^

### Randomisation and blinding

Participating schools will be randomly allocated to either the intervention or wait-list control condition at the school level by an independent statistician, not involved in the trial management, in accordance with International Council for Harmonisation of Technical Requirements for Pharmaceuticals for Human Use (ICH) Guidelines.^54^ A minimisation approach to randomisation^55 56^ will be used to balance key school characteristics across conditions (primary vs secondary school, rural vs urban location). Schools in the same region may train together or in a similar timeframe, in which case they will be randomly allocated to the same condition cluster, a priori, to avoid contamination risk. Due to the wait-list control design, participating schools, staff and students will be blinded to condition at the recruitment and consent stage only. Following randomisation, schools will be informed of their allocation to enable intervention scheduling and planning. Outcomes will be assessed via participant self-report, with no independent assessors. Data analysts will remain blinded to group allocation until primary analyses are complete by analysing de-identified datasets with masked group labels.

### Patient and public involvement

In this school-based trial, patient and public involvement refers to the involvement of students, families and school and education system staff rather than clinical patients. Public involvement occurs at multiple stages of study development and implementation.

The PFA-S programme was developed in response to and informed by NSW DoE staff consumer needs to better support children and adolescents in disasters. Well-being staff and teaching staff from one primary and one secondary school provided programme feedback following pilot deliveries, in preparation for rigorous testing.

NSW DoE Child Wellbeing and Mental Health Services staff experience and priorities informed the current research questions, choice of outcome measures and study design. A Staff Advisory Group (educators and well-being staff) and three Student Advisory Groups (ages 5–8, 9–12 and 13+ years) will be established prior to trial commencement to provide consultation across the study period.^57^ Advisory group members will be recruited with support from participating schools to ensure representation across demographic diversity, including socioeconomic status, gender, culture and rural communities. Advisory group involvement will be guided by participatory action principles^58–60^ to ensure that the study reflects the priorities, experiences and preferences of school communities. Structured workshops will be held to gather feedback on information materials and survey content (including developmental appropriateness), recruitment processes and strategies, interview and focus group protocols and implementation strategies. Staff and Student Advisory Groups and NSW DoE representatives will also be involved in shaping dissemination plans, including the format, language and accessibility of study findings for participating schools and related communities (eg, summary reports, presentations).

### Intervention

PFA-S was developed by members of the research team (EM and PK, see acknowledgements for full list) and NSW DoE in response to school staff expressing a need for improved capacity to support student well-being in disasters. Pilot deliveries to well-being staff, followed by teaching staff at one primary and secondary school, informed refinements to improve the acceptability and feasibility of the training.

The PFA-S programme is grounded in Hobfoll’s five principles for mass trauma management: promotion of safety, calm, self- and community-efficacy, connectedness and hope,^23^ with eight core actions, aligning with international guidelines,^26^ connect to the principles: contact and engagement, safety and comfort, stabilisation, information gathering of current needs and concerns, practical assistance, connection with social supports, providing information on coping and linking students with collaborative services. Training aims to assist staff to feel comfortable and confident to implement the five principles and eight actions of PFA and to build staff personal preparedness and self-care strategies to manage the demands of providing PFA in schools. The PFA-S programme includes education on the impacts of disasters on children across developmental stages, core PFA-S principles and actions, examples of disasters and serious incidents, and opportunities to practice applying PFA-S strategies to disaster-specific and school-specific example scenarios. Following training, staff may participate in school-based reflection sessions led by well-being staff. More details on programme content can be obtained by contacting the authors.

NSW DoE PFA-S master trainers will train existing well-being staff to become PFA-S facilitators for their region. Where possible, facilitators will be matched to schools based on local knowledge and experience. Facilitators will use a structured PFA-S Facilitator Manual to support consistent delivery across diverse school contexts while allowing for local community adaptations to match needs (eg, disaster examples, local supports, community connections). A sample of training sessions will be observed by researchers and developers to monitor adherence to core training components.^57^ Facilitators will be invited to complete post-training surveys including an implementation checklist and questions relating to engagement and suggested improvements.

Prior to training, staff participants will have the non-compulsory opportunity to review background reading and the PFA-S Participant Workbook. Two facilitators will deliver the training universally to all participating staff at the school, in either a single 2-hour block (face-to-face) or across two 1-hour sessions over 2 weeks (one online or face-to-face, one face-to-face). This format allows time to teach, apply, and reflect on core PFA-S principles and actions, represents a feasible commitment for busy staff (based on feedback during development), and aligns with departmental training timetable requirements. Training attendance (0, 1 or 2 hours) will be measured, with treatment adherence defined as full attendance to the two core training hours.

### Procedures

The trial will be conducted over two school years, from term 1, 2026 to term 4, 2027. Information and consent forms will be distributed to staff at participating schools via school leaders prior to trial commencement. Validated outcome assessment measures will be collected via surveys administered online using Qualtrics. Three outcome measure surveys (baseline, 1 month, 4 months) will take 20 min to complete. A postintervention implementation outcome survey will take 5 min to complete.

Figure 1 describes the study flow. All staff who provide consent will be invited to complete a baseline survey. Next, in intervention schools (n=9), the PFA-S training programme will be delivered universally to all participating staff, followed by a post-training evaluation survey and follow-up surveys at 1 month and 4 months. During the intervention period, wait-list control schools (n=9) will continue with usual classroom activities and have access to usual well-being supports. Staff will complete all scheduled surveys except the post-training evaluation survey. Following the 4-month follow-up, staff in wait-list control schools will be offered the PFA-S training programme.

**Figure 1:**
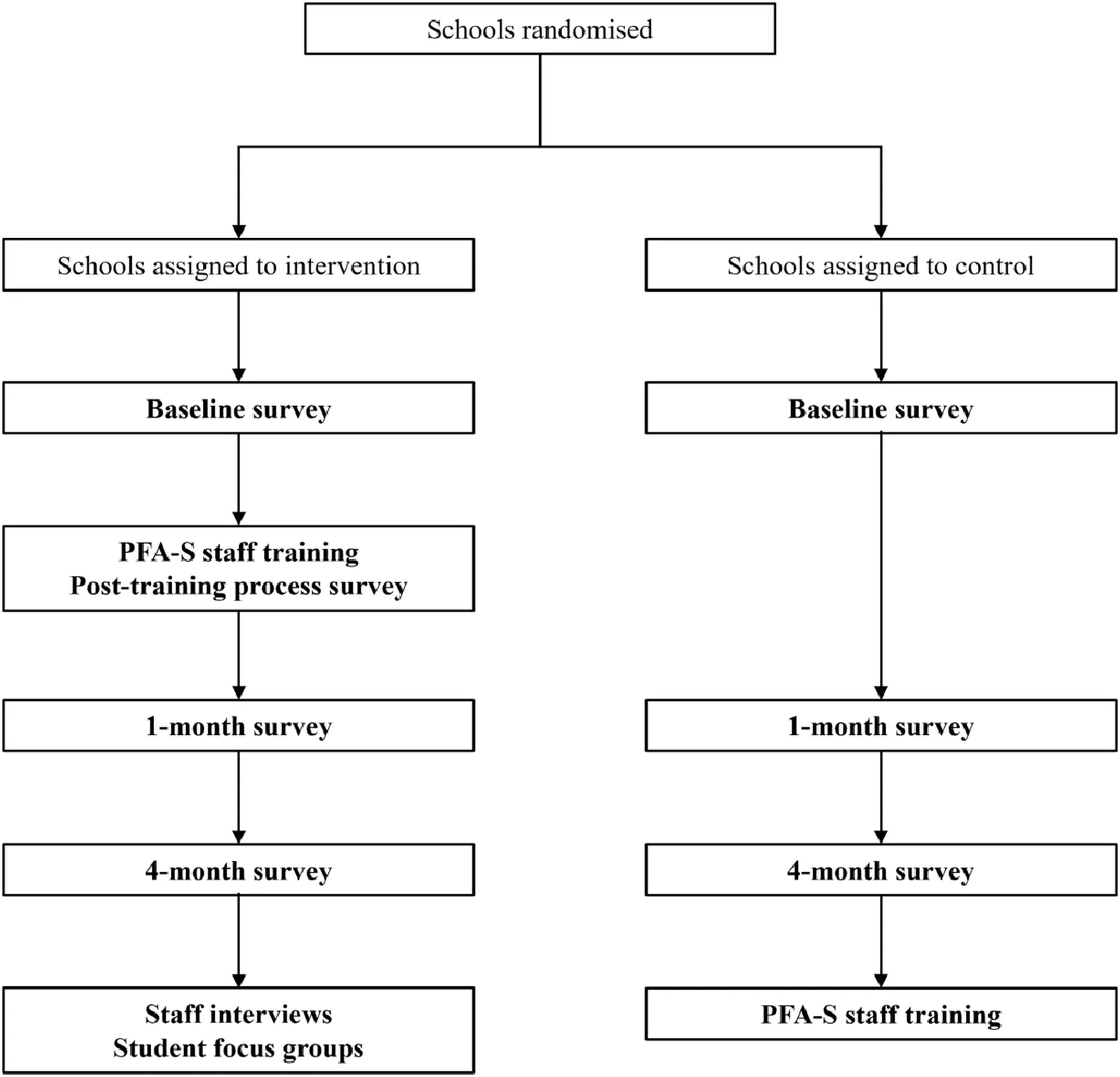
Participant flow chart. PFA-S, Psychological First Aid in Schools.

Students will not receive PFA-S training directly. They are considered potential end-users of improved staff support. Information and consent forms will be distributed to students and their parent/carer at participating schools via usual school communication channels prior to trial commencement. Students with parent/carer consent will provide assent through survey completion and, where applicable, through participation in focus groups.

All students aged ≥8 years at participating schools will be invited to complete outcome measures assessing their knowledge, experiences and perceptions of receiving PFA-S-style support, as well as unmet needs related to disaster preparedness, using surveys administered online via Qualtrics. Student surveys will be conducted at baseline, 1-month and 4-month follow-up, aligning with the teacher survey timeline. Researchers and school staff will assist students with survey completion by explaining instructions or reading questions aloud where required. An instructional video will also be provided to students to support understanding. Each survey will take about 20 min to complete.

Following the 4-month survey, 2–3 staff per intervention school will be invited to participate in a 30–60 min semistructured interview (in person or online via Zoom/Teams) to explore the acceptability, appropriateness and barriers and facilitators relating to potential and actual PFA-S use within their school culture and community (estimated n=20). In-person student focus groups (30–60 min) will be conducted to explore in more depth their experiences of receiving PFA-S style support and disaster preparedness needs within their school culture and community. Groups will include up to five students at a time from 9 to 12 selected schools, with approximately 15 students aged 5–8, 15 aged 9–12 and 15 aged 13 years and above (estimated n=45). Semistructured, open-ended questions will explore students’ disaster experiences and perceptions of staff responses, mapped to PFA-S principles.

Some schools may experience a disaster, a ‘near-miss’ (ie, a major weather event that threatened, but did not result in disaster), or another serious incident (eg, transport accident) during the trial. In such cases, with baseline data available, there will be a unique opportunity to examine the real-world application of PFA-S in a disaster. For affected schools, survey timeframes and measures will be dynamically adapted, and interviews and focus-groups will explore the perceived value, impacts, and limitations of PFA-S in a disaster context. The well-being of staff and students will be prioritised, and Advisory Groups and school leadership will be consulted to determine appropriate adjustments and participation expectations. In the event of a serious incident, a decision will be made about amending the study timeframe. Any adverse events relating to PFA-S training, PFA-S use, or research participation will be reported to the data safety and monitoring committee who will be responsible for stopping the research to prevent harm from occurring. Possible harms of the PFA-S intervention will be assessed via systematic negative changes in primary or secondary outcomes, and/or qualitative data.

### Measures

Table 1 presents the proposed measures and timeline for the PFA-S trial. Where available, reliable, validated and developmentally appropriate measures are used. Where these do not exist, bespoke measures have been designed. Consistent with co-design principles, all measures will be reviewed and pilot tested by the staff and student advisory groups before trial commencement and may be tailored accordingly.

**Table 1:** Survey measures and timeline

	Preintervention	Immediately post-training*	1 month post-intervention	4-month follow-up
Staff measures				
Demographic characteristics				
Age, gender, household income, postcode	✓			
Ethnicity, indigeneity	✓			
Role, years of experience	✓			
Mental health diagnoses	✓			
Preparedness				
PFA-S Knowledge, Attitudes and Skills^35 61^	✓		✓	✓
General Self-Efficacy 6-item^64^	✓		✓	✓
Mental health literacy, MHAC^65^	✓		✓	✓
Psychological preparedness, PPDTS AAM subscale^62^	✓		✓	✓
Mental health and well-being				
Well-being, WHO-5^66^	✓		✓	✓
Psychological distress, DQ5^68^	✓		✓	✓
Disaster experience				
Disaster exposure/severity, adapted^11 63^	✓		✓	✓
School leadership and culture	✓		✓	✓
School identification, SCASIM-St^69^	✓		✓	✓
Staff relations and leadership, adapted SCASIM-St^69^	✓		✓	✓
Student-staff relations, adapted Trusted Adults at School^70^	✓		✓	✓
Actual use				
Actual use, perceived value (bespoke)			✓	✓
Ongoing development (bespoke)			✓	✓
	Preintervention	Immediately post-training*	1 month postintervention	4-month follow-up
Implementation measures				
Fidelity, bespoke (facilitator)		✓		
Training attendance		✓		
Acceptability and satisfaction, adapted TFA (staff, leader)^77^		✓		
Implementation leadership, adapted ILS (staff, leader)^78^		✓		
Whole-school mental health promotion, SSPESH (leader)^79^		✓		
School context (leader)		✓		
Student measures				
Demographic characteristics				
Age	✓			
Gender	✓			
Mental health diagnoses (parent/carer-reported)	✓			
Mental health and well-being				
Well-being, SCWS^74^	✓		✓	✓
Anxiety, PROMIS^75^	✓		✓	✓
Depression, PROMIS^75^	✓		✓	✓
School leadership and culture				
Trusted adults at school^70 71^	✓		✓	✓
Peer Integration at School^70^	✓		✓	✓
School inclusion, SCASIM-ST^65^	✓		✓	✓
Help-seeking (ease/type)^72 73^	✓		✓	✓
Preparedness				
Psychological preparedness, adapted PPDTS AAM subscale^62^	✓		✓	✓
PFA-S experience, interest, future needs (bespoke)			✓	✓

* Intervention group only.

AAM, Anticipation, Awareness and Management; DQ5, Distress Questionnaire-5; ILS, Implementation Leadership Scale; MHAC, Mental Health Action Competencies; PFA-S, Psychological First Aid in Schools; PPDTS, Psychological Preparedness for Disaster Threat Scale; PROMIS, Patient Reported Outcomes Measurement Information System; SCASIM-ST, Australian School Climate and School Identification Measurement Tool; SCWS, Stirling Children's Wellbeing Scale; SSPESH, Survey of School Promotion of Emotional and Social Health; TFA, Theoretial Framework of Acceptability.

### Staff measures

#### Demographic characteristics

Demographic characteristics will be collected preintervention and will include age, gender, postcode, ethnicity, indigeneity, income, years of education experience, role type (selected from a list of relevant teaching, support, and well-being roles), current mental health and well-being responsibilities (Yes/No), and history of mental health diagnosis (depression, anxiety, PTSD, autism, attention-deficit/hyperactivity disorder (ADHD), other).

#### Primary and secondary outcomes

##### PFA-S preparedness (Knowledge, Attitudes and Skills)

The primary outcome is staff preparedness to deliver PFA-S, measured using an adapted PFA Knowledge, Attitudes and Skills scale (PFA-KAS),^35 61^ with additional bespoke items reflecting PFA-S content. This 15-item scale includes 4 knowledge items (eg, ‘I have a clear understanding of the role of PFA in disasters and crisis situations’), 3 attitude items (eg, ‘I feel well-prepared to use PFA with students in a disaster or crisis situation’) and 8 skill items aligned with the core PFA-S actions (eg, ‘In a disaster or crisis situation, I can identify signs that a student might need support for their well-being’, ‘I can apply strategies to comfort and calm students in distress’). Items are rated on a 5-point scale from 1 (strongly disagree) to 5 (strongly agree). Mean scores for each domain will be calculated, with higher scores reflecting better preparedness to deliver PFA-S.

##### Psychological preparedness

Psychological preparedness will be measured using the seven-item Anticipation, Awareness and Management (AAM) subscale of the Psychological Preparedness for Disaster Threat Scale (PPDTS16).^62^ Example items include: ‘I think I am able to manage my feelings pretty well in difficult and challenging situations’, ‘In a natural hazard/disaster situation I would be able to cope with my anxiety and fear’. Items are rated on a 4-point scale from 1 (not at all true of me) to 4 (exactly true of me). Scores will be summed and can range from 7 to 28, with higher scores reflecting greater preparedness. The original eight-item AAM subscale shows strong internal consistency (α=0.96), with confirmatory factor analysis supporting reduction to the seven-item version without compromising reliability in the Australian context.^62^

### Predictors and moderators

#### Disaster exposure

Disaster exposure will be measured using a scale adapted from the Life Events Checklist for Diagnostic and Statistical Manual of Mental Disorders, Fifth Edition (DSM-5)^63^ and previous research by Macleod *et al*.^11^ Participants will indicate if they have been previously impacted by specific events (bushfire, flood, severe storm, COVID-19, significant accident, assault or sexual assault, suicide or other sudden death, other very stressful events). For each event, impact is assessed categorically as: ‘I was personally impacted’, ‘My family member(s) or close friend(s) were impacted’, ‘My school community (staff, students, families) was impacted’, ‘No impact on me or anyone close to me’, ‘Prefer not to say’. For extreme weather events with personal impact (bushfire, flood, severe storm), participants will report the number of times impacted and rate overall severity on a 4-point scale from 1 (no impact) to 4 (severe). Severity ratings will be summed to provide an overall personal impact of extreme weather events score ranging from 0 to 12. Participants will also be asked to indicate if they have experience providing practical or emotional support to those affected by disasters.

In the follow-up survey only, participants who report personal impact from an extreme weather event during the trial will rate the severity of impact across nine life domains (mental health, physical health, personal property, personal relationships, community connection, capacity to work, financial stress, accommodation disruption, caring responsibility). Each domain is rated on a 4-point scale from 0 (no impact) to 3 (severe negative impact), with a not applicable option. Ratings will be summed to provide an overall impact score.

#### General self-efficacy

General self-efficacy will be measured using the 6-item General Self-Efficacy Short-Form; α=0.79 to 0.88.^64^ Items (eg, ‘No matter what comes my way, I’m usually able to handle it’) are rated on a 4-point scale from 1 (not at all true) to 4 (exactly true). Scores are summed and can range from 6 to 24, with higher scores representing greater self-efficacy.

#### Mental health literacy

Mental health literacy will be measured using six items from the adapted Mental Health Action Competencies scale.^65^ Items include ‘I know when it is appropriate to involve a specialist when dealing with a student with mental stress, disorder or illness’, ‘I know where to refer students with increased risk for mental stress, disorders or illnesses if necessary’. Items are rated on a 4-point scale from 1 (not at all true of me) to 4 (exactly true of me). A mean score is calculated, with higher scores indicating greater mental health literacy.

#### Psychological well-being

Psychological well-being will be measured using the WHO-5 well-being Index, α=0.89.^66^ The five items assess positive well-being over the past 2 weeks (eg, ‘I have felt calm and relaxed’, ‘I woke up feeling fresh and rested’), using a scale from 0 (at no time) to 5 (all of the time). The total score (0–25) is converted to a percentage (0–100). Higher scores indicate greater well-being and lower mortality risk.^67^

#### Psychological distress

Psychological distress will be measured using the Distress Questionnaire-5,^68^ a five-item scale assessing symptoms of psychological distress over the past month (eg, ‘my worries overwhelmed me’, ‘I felt hopeless’), α=0.86. Items are rated on a 5-point scale from 1 (never) to 5 (always) and summed (possible range –25), with higher scores indicating greater distress.

#### School identification

School identification will be measured using the six-item Identification subscale from the Australian School Climate and School Identification Measurement Tool (SCASIM-St), α=0.94.^69^ Example items include ‘I feel a strong connection with this school’ and ‘I care about this school’. Items are rated on a 5-point scale from 1 (strongly disagree) to 5 (strongly agree). A mean score is calculated, ranging from 1 to 5, with higher scores indicating stronger school identification.

#### Staff relations and leadership

Adapted items from the SCASIM-St climate subscales will assess staff relations (two items, eg, ‘Staff care about each other’) and perceptions of school leadership (two items, eg, ‘I have confidence in the leadership of this school (principal and executive team) at this school’) using the same 5-point scale, with mean scores calculated for each domain. Domain scores can range from 1 to 5.

#### Student-staff relations

Student-staff relations will be measured using four items adapted from the Trusted Adults at School scale (eg, ‘Staff listen to students’).^70^ Items are rated on a 5-point scale from 1 (strongly disagree) to 5 (strongly agree). Mean scores are calculated, with higher scores (range=1 to 5) indicating more positive perceptions of student–staff relationships.

#### Actual use of PFA-S (follow-up survey only)

Staff will report how often they have used PFA principles or actions to support students in the last month (0=not applicable or did not need to do this, 1=once, 2=2–3 times, 3=4–6 times, 4=7+ times). If staff report no use of PFA-S, they will be asked to select reasons for non-use (eg, ‘I do not know enough about PFA’, ‘I did not need to’, ‘It is not part of my professional role or responsibility’). Staff will also report how often they have used each of the four specific PFA-S skills adapted from the Skills domain of the PFA-KAS (eg, ‘Connected a student in distress with appropriate staff, peer, and/or family supports’) using the same scale.

#### Ongoing development (follow-up survey only)

Staff will be asked whether, since the last survey, they have engaged in any general disaster-related or mental health-related professional development. Intervention condition participants will also report whether they have engaged in PFA-S-related development activities (eg, ‘Rewatching video or webinar content’).

An open-ended question will be asked at follow-up to explore any challenges or negative experiences associated with training or delivery of PFA-S to students.

### Student measures

#### Demographic characteristics

Demographic characteristics will be collected preintervention and will include age, gender (boy, girl, please describe), grade, and mental health diagnosis (select from: Autism, ADHD, learning difficulties, depression, anxiety, other) (parent/carer-report for students under 16).

#### School connection and culture

School identification will be measured using the six-item Identification subscale from the SCASIM-St, consistent with the staff survey. Peer integration at school will be assessed using four items designed to capture students’ sense of connection at school (eg, ‘I have many friends’).^70^ Trusted adults at school will be measured using a four-item scale to assess students’ perceptions of adult support at school, α=0.90 (66) (eg, ‘At my school there is an adult who listens to me’).^70 71^ All items are rated on a 5-point scale from 1 (strongly disagree) to 5 (strongly agree). Mean scores will be calculated for each domain, with higher scores reflecting stronger school identification, peer integration and trusted adults at school.

#### Help-seeking

Overall help-seeking confidence will be measured with a single item adapted from^72^: ‘If you were stressed or upset, how easy would it be for you to get help?’. Responses are rated on a 5-point scale from 0 (not at all easy) to 5 (extremely easy). Help-seeking intentions by source will be assessed using a 5-item adaptation of the General Help Seeking Questionnaire.^73^ Students rate how likely they would be to seek help from different sources if stressed or upset: 1=An adult at my school, 2=A friend or peer at my school, 3=A parent or caregiver, 4=A person, group, or organisation in my community, 5=Someone else (open text). Each item is rated on a 7-point scale from 1 (extremely unlikely) to 7 (extremely likely). Mean scores will be calculated for each help source, with higher scores indicating greater likelihood of seeking help. Open-text responses will be analysed qualitatively.

#### Psychological preparedness

Psychological preparedness will be measured using the 7-item AAM sub-scale of the PPDTS16,^62^ adapted for students with minor language modifications (eg, ‘I can manage my feelings pretty well in difficult situations’, ‘In a disaster I would be able to cope with my anxiety or fear’). Four additional items developed for this study will assess students’ self-efficacy to support others in stressful situations (eg, ‘I could tell if friends or family were stressed or having a hard time in a stressful situation’, ‘I know what I can do to help my friends, classmates and family stay calm and well’). Items are rated on a 5-point scale from 1 (strongly disagree) to 5 (strongly agree). Scores are summed for each domain, with higher scores reflecting greater preparedness or self-efficacy to provide support.

#### Well-being

Well-being will be assessed using the Stirling Children’s Wellbeing Scale,^74^ excluding four lie-scale items. The 12-item scale includes statements about mood, enjoyment, and positive expectations (eg, ‘I’ve been feeling calm’). Items are rated on a 5-point scale from 1 (never) to 5 (all the time), α=0.82. Total scores range from 12 to 60, with higher scores reflecting greater emotional well-being.

#### Depression and anxiety

Depression and anxiety will be measured using two subscales (anxiety and depressive symptoms) from the Patient Reported Outcomes Measurement Information System Pediatric-25 Profile v.3.0.^75^ Both subscales have shown high internal consistency (α>0.90).^76^ Each subscale includes four items assessing symptoms over the past 7 days (eg, ‘I felt sad’, ‘I felt nervous’). Items are rated on a 5-point scale from 1 (never) to 5 (almost always). Subscale scores are calculated by summing item scores, with higher scores indicating greater levels of depression or anxiety.

#### PFA-S experience

Students will be asked whether they received help from an adult at school when they were stressed or upset in the last month (Yes/No). If Yes, they will rate how helpful the adult was on a scale of 1–5 and select from a list of support actions received (eg, ‘Helped to comfort or calm me’, ‘Helped me to get support from an adult or group outside of school’). If No, they will select reasons for not receiving help (eg, ‘I didn’t need help’, ‘I didn’t think an adult at school would help’). Responses will be analysed descriptively and used to explore student experiences of PFA-style support.

#### PFA-S interest

PFA-S interest will be assessed with four items: ‘I would like to know more about what I can do to help myself/my friends/my classmates/my family stay calm and well in a disaster’. The items will be rated on a 5-point scale from 1 (strongly disagree) to 5 (strongly agree).

#### Disaster preparedness education needs

Disaster preparedness education needs will be assessed with an open-text question: ‘In your own words, what would you like to know more about for dealing with disasters?’. Responses will be analysed qualitatively to identify themes related to unmet needs and interests in disaster preparedness and well-being.

### Implementation measures

#### Fidelity of training delivery

After each training session, facilitators will complete a fidelity checklist to self-assess adherence to training content and five bespoke survey questions relating to PFA-S value (‘PFA-S training is valuable for school staff’), staff engagement (eg, ‘Staff engaged well with the online content’), and facilitator preparedness (eg, ‘I was prepared to deliver the PFA-S content’). Items are rated on a 5-point scale from 1 (strongly disagree) to 5 (Strongly agree). Facilitators will also be asked about any events of distress, how they were managed, and provide any other open-text feedback and suggestions. Responses will be used to assess fidelity and identify areas for improvement.

#### Acceptability and satisfaction

Staff will complete a post-training survey including the adapted Intervention Satisfaction and Discontinuation: Theory of Acceptability scale (Theoretial Framework of Acceptability; TFA).^77^ Seven items assess appeal, burden, perceived effectiveness, intervention coherence, self-efficacy, opportunity cost, and general acceptability (eg, ‘Did you like or dislike the PFA-S training programme?’, ‘How much effort did it take to participate in the PFA-S training programme?’). Three additional bespoke items will assess relevance to role, facilitator effectiveness, and endorsement of the programme. Items are rated on 5-point scales tailored to each question. Two items (burden, opportunity costs) are reverse scored. A total mean score is calculated, ranging from 1 to 5, with higher scores indicating greater acceptability of PFA-S training. Three open-ended questions will explore programme strengths and areas for improvement.

#### Staff perceptions of implementation leadership

Staff will also complete two items from the staff-report version of the Implementation Leadership Scale (ILS)^78^: ‘At my school there is leadership to support staff efforts to learn more about PFA-S’, ‘At my school there is leadership to support staff efforts to use PFA-S’. Items are rated on a 5-point scale from 0 (not at all) to 4 (to a very great extent), and a mean score is calculated.

#### Implementation leadership

School leaders (eg, principals, executive staff) overseeing PFA-S implementation will complete either a survey or structured interview based on the following measures. The ILS^78^ is a 12-item scale assessing the degree to which a leader views themselves as Proactive (3 items), Knowledgeable (3 items), Supportive (3 items) and Perseverant (3 items) in regard to PFA-S implementation, α=0.98. Items are rated on a 5-point scale from 0 (not at all) to 4 (to a very great extent). Mean scores are calculated for each subscale and the total scale. Five bespoke items will assess leadership satisfaction and perceptions of staff buy-in (eg, ‘Staff engaged well in PFA-S training’, ‘I am satisfied with the PFA-S training’), rated on a 5-point scale from 1 (strongly disagree) to 5 (strongly agree). Open-ended questions will explore strengths, barriers and any unintended consequences of implementation.

#### Whole-school mental health promotion

School leaders will also complete the Survey of School Promotion of Emotional and Social Health, ^79^which includes 13 items across four domains: (a) creating a positive school community, (b) teaching social and emotional skills, (c) engaging the parent community and (d) supporting students experiencing mental health difficulties. Items are rated on a 4-point scale: 0=not yet in place, 1=introducing, 2=taking hold, 3=completely in place. Domain scores and a total score (possible range 0–39) will be calculated.

#### School context

To understand contextual factors influencing implementation, researchers will collect school leader reports of community disaster exposure, as well as publicly available data on school decile, size, gender distribution and other characteristics.

#### Staff interviews

At 4-month follow-up, semistructured interviews will be conducted with intervention condition teachers and well-being staff to explore acceptability and satisfaction with PFA-S programme content, and perceived barriers and facilitators to using PFA-S to support students during stressful situations. If a specific disaster or ‘near-miss’ has occurred within the school community, staff will also be asked to reflect on how PFA-S prepared them to support students, application of PFA-S principles and actions, and remaining unmet needs for student support.

### Student focus groups

At 4-month follow-up, focus groups will be conducted with approximately five students from each school, prioritising those in the intervention condition. Using open-ended prompts, students will be asked to discuss experiences of PFA-S supports and principles, perceived strengths and weaknesses of PFA-S principles, and current gaps and needs in mental health supports for students. If a disaster or ‘near-miss’ has occurred, students will also be asked to share their views on how staff supported students, their experiences of PFA-S actions from staff, and any remaining unmet needs for support.

### Data analysis plan

For all bespoke measures, we will determine reliability of the scale using Cronbach’s alpha and test–retest reliability. We will also assess the unidimensionality of each scale and consider convergent and divergent validity, comparing scores against the included validated measures of psychological preparedness, self-efficacy, mental health literacy, and mental health symptoms.

Quantitative analyses will follow an intention-to-treat (ITT) approach. The ITT population comprises all eligible participants from schools randomised to either study condition who provide study data at one or more assessment timepoints, regardless of adherence to the intervention or withdrawal from the study. Participants will be analysed according to the randomised condition assigned to their school. Participants who transfer between participating schools during the study will remain assigned to the allocation of the first school at which they enrolled in the study. Participants who join a participating school post-randomisation will be analysed according to the allocation of that school. An exploratory per-protocol sensitivity analysis will be conducted, excluding participants who disclose contamination (eg, incidental exposure to PFA learning), move schools, or fail to attend the minimum threshold of the 2-hour training session. If attrition exceeds our estimates, we will conduct a completer analysis to investigate the impact of attrition on the outcomes.

For the primary and secondary outcomes, mixed-model repeated measures (MMRM) analyses will be used. MMRM accommodates missing data without relying on biased techniques, such as last observation carried forward. By incorporating random effects for each school, MMRM also accounts for clustering and allows assessment of the strength and significance of clustering effects. This method is widely regarded as the most robust approach for analysing cluster randomised trials.^80 81^ Sensitivity, subgroup and exploratory analyses will be conducted using MMRM to examine changes in exploratory outcomes and identify potential mediators and moderators of training effectiveness, such as school culture, individual mental health, disaster experience, demographic characteristics (eg, age, gender). Dichotomous exploratory outcomes will be analysed using binary mixed models, which are analogous to linear MMRM.

Qualitative data from staff interviews and student focus groups will be analysed using Framework Analysis,^82^ a method well-suited to applied health research.^83^ This systematic approach involves either inductive or deductive coding and the development of a matrix of themes across participants, facilitating collaborative interpretation and analysis.

### Monitoring

Oversight of trial conduct, participant safety, and data integrity will be maintained by an internal data safety and monitoring committee comprising the lead researcher, clinical psychologists, the data manager and staff liaison. The committee will review adverse events, participant safety issues, trial conduct, and data integrity and report to the wider project guidance research team at regular meetings. Committee members are independent of the study funder, and the funder will not have a role in monitoring data or trial conduct. A formal committee charter has not been developed due to the low-risk nature of the intervention.

## Ethics and dissemination

The research has received relevant ethical and education department approvals (ANU Human Research Ethics Committee: 2025 0018; NSW DoE State Education Research and Partnerships: 3544). All research data will be securely stored electronically at the Australian National University for a minimum of 5 years following publication of any research outputs and will only be accessible to members of the research team. After this period, electronic survey and transcription data will be archived in a deidentified format (all reasonably identifying information removed) and may be shared with other researchers on request, subject to approval by the original research team.

Following the trial, study findings will be communicated to participating schools through summary reports and presentations, which schools may share with their broader communities. Plain-language summaries will be developed to support accessibility for non-academic audiences. Academic dissemination will occur through publications in peer-reviewed journals and conference presentations. Broader public dissemination will be supported through a project website and social media. Study findings will also be used to inform improvements to the PFA-S programme and to guide future research and implementation strategies.

## Discussion

This study will be the first rigorous evaluation of PFA-S in the Australian school context. It addresses a critical gap in the evidence base for school-based disaster mental health interventions, particularly considering the increasing frequency and severity of climate-related disasters and the associated impacts on youth. The results of this study will provide vital new evidence on the feasibility, acceptability and effectiveness of a disaster mental health prevention programme to prepare school staff to protect students from the negative impacts of disasters and other serious incidents.

Key anticipated outcomes include (a) the development of a feasible and effective disaster preparedness and response training programme for school staff; (b) the development of a pathway for integration and sustainable delivery in schools; (c) insights into the contextual factors influencing programme implementation and effectiveness; (d) a deeper understanding of student perspectives and unmet needs and (e) evidence for intervention effectiveness to support policy and funding decisions and promote nationwide uptake. Potential limitations include potential selection bias in school, staff and student participation, which may limit representativeness across geographic or socioeconomic contexts.

This study will provide valuable new knowledge on PFA training interventions that can support the well-being and mental health of children in natural disaster contexts. PFA-S has the potential to be delivered in other Australian states and internationally, and to be adapted to other settings, providing an accessible intervention to promote child and adolescent mental health and well-being in disasters. Furthermore, data from students will be used to inform next steps in the research, focusing on developing peer PFA training and any other indicated disaster supports, that target children and adolescents directly.

## Supplementary material

10.1136/bmjopen-2026-121438 online supplemental file 1
