# Supplementary material for: Psychological First Aid in Schools to prepare staff for supporting student mental health in disasters and critical incidents: protocol for a cluster-randomised controlled trial
Source: BMJ Open. 2026 Nov 11;16(11):e121438. doi: 10.1136/bmjopen-2026-121438 (PMC13756367; doi:10.1136/bmjopen-2026-121438)
Supplement: Supplementary file 1 [file bmjopen-2026-121438.reviewer_comments.pdf]

PEER REVIEW HISTORY

BMJ Open publishes all reviews undertaken for accepted manuscripts. Reviewers are asked to complete a checklist review form and are provided with free text boxes to elaborate on their assessment. These free text comments are reproduced below.

ARTICLE DETAILS

Title (Provisional)

Psychological First Aid in Schools to prepare staff for supporting student mental health in disasters and critical incidents: protocol for a cluster-randomised controlled trial

Authors

Macleod, Emily; Curll, Sonia; Batterham, Philip J; Cruwys, Tegan; Farrer, Lou; Goodyear, Melinda; Heffernan, Timothy; Kostelas, Pauline; Morgan, Bradley; Morse, A.; McCallum, Sonia; Pasalich, Dave S; Rogers, Marg; Rodney, Rachael; Turley, Bree; Calear, Alison L.

VERSION 1 - REVIEW

Reviewer 1

Name Mundagowa, Paddington

Affiliation University of South Carolina Arnold School of Public Health, Epidemiology and Biostatistics

Date 30-May-2026

COI None

Thank you for planning to partake this important work. The objectives, methdods and significance of the protocol manuscript were well articulated. These are some minor comments for your consideration.

1. I suggest defining the term disaster in the context of this study in the first paragraph. Despite the term being partly defined in Lines 130-131, moving it up will prime the reader on which ‘disasters the authors refer to.

2. Line 98: Authors could consider deleting the word ‘but’ to enhance clarity.

3. Line 401: Kindly provide the name of the scale to be used or at least mention the authors for references 58 and 59.

Reviewer 2

Name Haldar, Partha

Affiliation **All India Institute of Medical Sciences New Delhi, Centre for Community Medicine, Old OT Block**

Date **11-Jul-2026**

COI **None**

This is a well-organized and largely SPIRIT-compliant protocol, with genuine strengths (patient/public involvement, mixed-methods implementation evaluation, inclusion of child/adolescent voice, pre-registered design). The following comments focuses on gaps, ambiguities, and methodological vulnerabilities

Population definition: The only population definition given is in the Data Analysis Plan (page 27): "Quantitative analyses will follow an intent-to-treat approach, including all participants randomised, regardless of adherence to the intervention or withdrawal from the study." That's it. There is no per-protocol (or as-treated / complier-average causal effect) analysis specified anywhere in the protocol — not as the primary analysis, not as a sensitivity analysis, not even mentioned as a future possibility.

-The ITT definition itself is under-specified for a cluster trial. Randomization occurs at the school level, but outcomes are collected and analyzed at the individual (staff/student) level. A proper cluster-trial ITT statement needs to specify that individuals are analyzed according to the allocated condition of their school, regardless of their personal exposure to training — the current wording ("all participants randomised... regardless of adherence") reads as if individuals themselves were randomized, which isn't quite accurate here and should be corrected for clarity (SPIRIT Item 27b explicitly asks for "in which group").

-There is real, plausible variability in individual "dose" that ITT will mask. This is not a simple drug-vs-placebo trial. The intervention involves:

• A 2-hour session or a split format (1 hour online/face-to-face + 1 hour face-to-face, across two weeks) — meaning some staff could attend one session but not the other.

• Delivery scheduled at each school's convenience within the recruitment window — normal staff absence, leave, or scheduling conflicts could easily produce partial attendance.

• An "actual use" outcome that the authors themselves collect at follow-up (how often staff report using PFA-S skills), which is essentially an implicit acknowledgment that exposure to the intervention's effects will vary even among trained staff.

-With no per-protocol or dose-based analysis, a null or attenuated ITT result becomes hard to interpret: it could mean PFA-S doesn't work, or it could mean many staff received a diluted dose of it. A per-protocol or CACE analysis (even as a secondary/sensitivity analysis, properly labeled as non-causal/exploratory if per-protocol) would help distinguish these explanations — and its complete absence limits what the trial can ultimately tell readers about efficacy versus effectiveness-under-imperfect-implementation.

-No adherence definition exists to even build a per-protocol population. Even if the authors wanted to add one post hoc, the protocol doesn't pre-specify what would count as "adherent" — e.g., attendance at both sessions, a minimum proportion of session content, completion of the (non-compulsory) pre-training workbook, or some threshold on the facilitator fidelity checklist. Without this pre-specification, any later per-protocol analysis would risk being a post-hoc, data-driven definition — exactly the kind of flexibility prospective registration is meant to prevent.

-Post-randomization entrants are not addressed. The trial runs across two full school years (Term 1 2026–Term 4 2027). Staff who are hired, or students who transfer in, after their school's randomization has occurred are not accounted for in the population definition — are they eligible for baseline-equivalent enrollment mid-study, and if so, how are they folded into an "as randomised" ITT population that by definition didn't include them at baseline? This is unaddressed.

- The Primary outcome measure seems largely unvalidated. The PFA-KAS is described as "adapted" from two prior instruments "with additional bespoke items reflecting PFA-S content." No pilot psychometric data (internal consistency, factor structure) for this specific 15-item adapted version are reported. Since this is the primary outcome for the trial's central hypothesis, this is a substantive measurement-validity gap. The same applies to numerous other "bespoke" instruments used throughout (actual-use items, PFA-S experience/interest items, leadership satisfaction items, facilitator fidelity items).. self-developed, unvalidated measures are used extensively across both primary and secondary/exploratory outcomes. Authors may please give further details for same.

- Despite the Introduction's explicit warning about potential harms from universal school MH interventions, the only harm-detection mechanism described is an open-ended question to staff about "challenges or negative experiences." Why there is no equivalent systematic harms assessment for students (the more vulnerable population) is confusing.

- SPIRIT Item 12 asks for the trial's framework (superiority/equivalence/non-inferiority/exploratory) to be stated explicitly. The protocol is implicitly a superiority/effectiveness-implementation hybrid design but this label is never used.

- Sample size: Power calculation covered only the staff related primary outcome; no justification is given for secondary, exploratory, or any student-level outcome. Given that the student sample (~1,260) is analyzed for wellbeing, anxiety/depression, help-seeking, and PFA-S-experience outcomes, the complete absence of any power calculation for this seems conspicuous. Readers cannot judge whether the trial is adequately powered to detect anything meaningful in students, who are arguably the population in whome the intervention is ultimately meant to act/benefit.

- Randomization and allocation-concealment procedures are appropriately described (independent statistician, minimization).

- Blinding is inherently minimal (open-label by design, given the wait-list structure), and this is transparently acknowledged. However, the protocol does not discuss contamination risk — staff or students moving between schools, shared regional professional-development forums, or informal information exchange within the same state education system could plausibly contaminate the control arm's exposure to PFA-S content, and no contamination-monitoring or sensitivity analysis is planned.

- Attrition assumption ("expected to be low... due to high departmental buy-in") is very optimistic. While the trial is expected for two full school years, it may be prudent to that authors consider this as a sensitivity analysis.

- No interim analysis or stopping rules are specified (marked N/A), which is plausible for a low-risk behavioral/training intervention but could still be considered given the acknowledged potential for participant distress if a genuine disaster occurs mid-trial.

- SPIRIT Item 5: no separately accessible Statistical Analysis Plan is provided. Given the large number of primary, secondary, exploratory, predictor/moderator, and implementation outcomes (Table 1 lists well over 20 measures across two respondent groups and four time points), the absence of a separately timestamped SAP is a meaningful gap.

- Item 9b analogue: no rationale given for comparator choice. The self-completed checklist marks Item 9b ("explanation for choice of comparator") as "na." A wait-list control is a defensible and common choice for a low-risk school intervention, but SPIRIT explicitly requires this be justified, not marked not-applicable — the rationale (ethical equipoise, eventual access, feasibility) should be stated rather than assumed.

VERSION 1 - AUTHOR RESPONSE

Reviewer 1:
1. I suggest defining the term disaster in the context of this study in the first paragraph. Despite the term being partly defined in Lines 130-131, moving it up will prime the reader on which 'disasters' the authors refer to.

We thank Reviewer 1 for the suggestion. We have expanded our definition of disasters and moved it up to the first paragraph of the introduction (p4):

> **"Disasters are events or series of events with consequences that overwhelm the capacity of the community to cope and give rise to secondary stressors and adversities [1-2]. In this paper, the term "disasters" is used inclusively to encompass climate-related events, such as extreme weather events and earthquakes, and other critical incidents, including transport accidents, acts of violence, and sudden deaths affecting a community."**

2. Line 98: Authors could consider deleting the word 'but' to enhance clarity.

We have deleted as suggested (p5).

3. Line 401: Kindly provide the name of the scale to be used or at least mention the authors for references 58 and 59.

We have made the following edits as suggested (p18):

> **"Disaster exposure will be measured using a scale adapted from <u>the Life Events Checklist for DSM-5 [LEC-5]</u> [63] and <u>previous research by Macleod et al.</u> [64]."**

Reviewer 2:
This is a well-organized and largely SPIRIT-compliant protocol, with genuine strengths (patient/public involvement, mixed-methods implementation evaluation, inclusion of child/adolescent voice, pre-registered design). The following comments focuses on gaps, ambiguities, and methodological vulnerabilities

We thank Reviewer 2 for the support and considerations for strengthening the protocol. Our responses follow each of the Reviewer 2's suggestions below.

1. Population definition: The only population definition given is in the Data Analysis Plan (page 27): "Quantitative analyses will follow an intent-to-treat approach, including all participants randomised, regardless of adherence to the intervention or withdrawal from the study." That's it. There is no per-protocol (or as-treated / complier-average causal effect) analysis specified anywhere in the protocol — not as the primary analysis, not as a sensitivity analysis, not even mentioned as a future possibility.

Points 1 and 2 are addressed together below.

2. The ITT definition itself is under-specified for a cluster trial. Randomization occurs at the school level, but outcomes are collected and analyzed at the individual (staff/student) level. A proper cluster-trial ITT statement needs to specify that individuals are analyzed according to the allocated condition of their school, regardless of their personal exposure to training — the current wording ("all participants randomised… regardless of adherence") reads as if individuals themselves were randomized, which isn't quite accurate here and should be corrected for clarity (SPIRIT Item 27b explicitly asks for "in which group").

We appreciate the comments and suggestions to improve the definitions, clarify that schools rather than individuals are randomised, and include a per-protocol analysis. To address reviewer points 1 & 2, we have added the following to the data analysis plan (p28-29):

> **"The ITT population comprises all eligible participants from schools randomised to either study condition who provide study data at one or more assessment time points, regardless of adherence to the intervention or withdrawal from the study. Participants will be analysed according to the randomised condition assigned to their school. Participants who transfer between participating schools during the study will remain assigned to the allocation of the first school at which they enrolled in the study. Participants who join a participating school post-randomisation will be analysed according to the allocation of that school. An exploratory per-protocol sensitivity analysis will be conducted, excluding**

participants who disclose contamination (e.g., incidental exposure to PFA learning), move schools, or fail to attend the minimum threshold of the 2-hour training session."

3. There is real, plausible variability in individual "dose" that ITT will mask. This is not a simple drug-vs-placebo trial. The intervention involves:
• A 2-hour session or a split format (1 hour online/face-to-face + 1 hour face-to-face, across two weeks) — meaning some staff could attend one session but not the other.
• Delivery scheduled at each school's convenience within the recruitment window — normal staff absence, leave, or scheduling conflicts could easily produce partial attendance.
• An "actual use" outcome that the authors themselves collect at follow-up (how often staff report using PFA-S skills), which is essentially an implicit acknowledgment that exposure to the intervention's effects will vary even among trained staff.
-With no per-protocol or dose-based analysis, a null or attenuated ITT result becomes hard to interpret: it could mean PFA-S doesn't work, or it could mean many staff received a diluted dose of it. <u>A per-protocol or CACE analysis (even as a secondary/sensitivity analysis, properly labeled as non-causal/exploratory if per-protocol) would help distinguish these explanations — and its complete absence limits what the trial can ultimately tell readers about efficacy versus effectiveness-under-imperfect-implementation.</u>
-No adherence definition exists to even build a per-protocol population. Even if the authors wanted to add one post hoc, <u>the protocol doesn't pre-specify what would count as "adherent"</u> — e.g., attendance at both sessions, a minimum proportion of session content, completion of the (non-compulsory) pre-training workbook, or some threshold on the facilitator fidelity checklist. Without this pre-specification, any later per-protocol analysis would risk being a post-hoc, data-driven definition — exactly the kind of flexibility prospective registration is meant to prevent.

Thank you for these helpful observations and detailed notes. In our survey we collect data to determine training attendance. We apologise for omitting this information from the protocol. We have added (p14):

> **"Training attendance (0, 1, or 2 hours) will be measured, with treatment adherence defined as full attendance to the two core training hours."**

Training attendance is also now included in Table 1 Survey Measures and Timeline (p28).

We have also amended the analysis plan to include an exploratory per-protocol analysis that accounts for incomplete exposure to the intervention (see response to points 1&2 above).

4. Post-randomization entrants are not addressed. The trial runs across two full school years (Term 1 2026–Term 4 2027). Staff who are hired, or students who transfer in, after their school's randomization has occurred are not accounted for in the population definition — are they eligible for baseline-equivalent enrolment mid-study, and if so, how are they folded into an "as randomised" ITT population that by definition didn't include them at baseline? This is unaddressed.

Thank you for highlighting this missing information. We have now clarified that, because schools rather than individuals are randomised, staff and students who enter a participating school post-randomisation remain eligible for study participation (see response to points 1&2 above). Participants who join after baseline data collection may contribute data at subsequent assessment timepoints and will be included in the ITT population and analysed according to the randomised allocation of their school. To further clarify this in the protocol, we have added (p10):

> **"Participants at each randomised school who do not complete baseline assessment remain eligible to enrol at subsequent assessment time points."**

5. The Primary outcome measure seems largely unvalidated. The PFA-KAS is described as "adapted" from two prior instruments "with additional bespoke items reflecting PFA-S content." No pilot psychometric data (internal consistency, factor structure) for this specific 15-item adapted version are reported. Since this is the primary outcome for the trial's central hypothesis, this is a substantive measurement-validity gap. The same applies to numerous other "bespoke" instruments used throughout (actual-use items, PFA-S experience/interest items, leadership satisfaction items, facilitator fidelity items).. self-developed, unvalidated measures are used extensively across both primary and secondary/exploratory outcomes. Authors may please give further details for same.

On p16, we explain:

> **"Where available, reliable, validated, and developmentally appropriate measures are used. Where these do not exist, bespoke measures have been designed. Consistent with co-design principles, all measures will be reviewed and pilot tested by the staff and student advisory groups before trial commencement and may be tailored accordingly."**

Unfortunately, no validated measures exist that can be used to test the core assumptions and knowledge relating to PFA-S training. The adapted measure is based on a robust and well-established framework for assessing learning outcomes in applied psychology (the KAS model). However, we acknowledge the limitation of using an untested scale and have taken the following steps to mitigate the risk.

For all bespoke measures we will use advisory group testing to determine face-validity and user ease. We have also added the following to the data analysis plan (p28):

> **"For all bespoke measures, we will determine reliability of the scale using Cronbach's alpha and test-retest reliability. We will also assess the unidimensionality of each scale and consider convergent and divergent validity, comparing scores against the included validated measures of psychological preparedness, self-efficacy, mental health literacy, and mental health symptoms."**

6. Despite the Introduction's explicit warning about potential harms from universal school MH interventions, the only harm-detection mechanism described is an open-ended question to staff about "challenges or negative experiences." Why there is no equivalent systematic harms assessment for students (the more vulnerable population) is confusing.

Thank you for highlighting the importance of considering risk of harm. Given that students do not directly receive PFA-S training or education, it would be difficult to identify a specific, relevant open-ended question to match the staff question. The student survey does, however, explicitly ask students about types of help provided by staff, and staff helpfulness, which then allows students to identify any harmful actions resulting from PFA as "unhelpful." Further, harms will also be able to be detected for both staff and students if any systematic negative changes in primary and/or secondary outcome measures are observed. We have added to the protocol (p16):

> **"Possible harms will be assessed via systematic negative changes in primary or secondary outcomes, and/or qualitative data."**

7. SPIRIT Item 12 asks for the trial's framework (superiority/equivalence/non-inferiority/exploratory) to be stated explicitly. The protocol is implicitly a superiority/effectiveness-implementation hybrid design but this label is never used.

The protocol already identifies the study as a type 1 hybrid implementation-effectiveness design; however, we agree that the trial framework should be stated explicitly to align with SPIRIT Item 12. We have modified the design sentence as follows (p9):

> **"A two-arm cluster-randomised controlled trial (RCT) using a type 1 hybrid implementation-effectiveness design [45] <u>and a superiority framework</u> will be conducted across 18 public primary and secondary schools, with schools randomly allocated <u>in a 1:1 ratio</u> to either the intervention group (*n*=9) or a wait-list control group (*n*=9)."**

8. Sample size: Power calculation covered only the staff related primary outcome; no justification is given for secondary, exploratory, or any student-level outcome. Given that the student sample (~1,260) is analyzed for wellbeing, anxiety/depression, help-seeking, and PFA-S-experience outcomes, the complete absence of any power calculation for this seems conspicuous. Readers cannot judge whether the trial is adequately powered to detect anything meaningful in students, who are arguably the population in whom the intervention is ultimately meant to act/benefit.

We appreciate Reviewer 2's point. To clarify, the trial is designed to test whether the intervention enhances school preparedness to support students in a disaster, not to test student mental health in response to a disaster. The aims and hypotheses stated for students' outcomes are exploratory (see "Exploratory Aims," p9). Power calculations are not appropriate for exploratory outcomes as there is no hypothesis about any specific associations (see, Haile, 2023 "In exploratory research, there is no predefined hypothesis about an effect or

relationship. For this reason, statistical power is not a primary concern. In exploratory studies, the sample size is often driven by other factors, such as the availability of resources and other practical considerations, and the researcher is not required to perform a formal power analysis." See also Ko & Lim, 2021). The inclusion of a power calculation for exploratory outcomes would be an inconsistency with the trial design. We have added to the protocol (p11):

> **"Power calculations were not conducted for exploratory outcomes (Haile, 2023; Ko & Lim, 2021)."**

Regarding the secondary outcome, we have added the following (p10):

> **"For the secondary outcome, general psychological preparedness for disaster, there are no prior analogous school trials predicting effect sizes. Given some conceptual overlap between the primary and secondary outcome, we anticipate similar effect sizes for both primary and secondary outcomes."**

9. Randomization and allocation-concealment procedures are appropriately described (independent statistician, minimization).
Blinding is inherently minimal (open-label by design, given the wait-list structure), and this is transparently acknowledged. However, the protocol does not discuss contamination risk — staff or students moving between schools, shared regional professional-development forums, or informal information exchange within the same state education system could plausibly contaminate the control arm's exposure to PFA-S content, and no contamination-monitoring or analysis is planned.

This is a good point, thank you. We have survey items in the follow-up surveys that check for exposure to PFA-S content, formally or informally. We have amended our protocol and survey to include these checks for control participants as well. The following details for managing contamination risk have been added to the protocol:

> **"Schools in the same region may train together or in a similar timeframe, in which case they will be randomly allocated to the same condition cluster, a priori, to avoid contamination risk." (p11)**
> **"An exploratory per-protocol sensitivity analysis will be conducted, excluding participants who disclose contamination (e.g., incidental exposure to PFA learning), move schools, or fail to attend the minimum threshold of the 2-hour training session." (p30)**

10. Attrition assumption ("expected to be low… due to high departmental buy-in") is very optimistic. While the trial is expected for two full school years, it may be prudent to that authors consider this as a sensitivity analysis.

We have accounted for up to 20% attrition; this conservative approach aligns with previous studies (Beames et al., 2023), and we have added this reference to the protocol. We have also added the following (p29):

> **"If attrition exceeds our estimates, we will conduct a completer analysis to investigate the impact of attrition on the outcomes."**

11. No interim analysis or stopping rules are specified (marked N/A), which is plausible for a low-risk behavioural/training intervention but could still be considered given the acknowledged potential for participant distress if a genuine disaster occurs mid-trial.

Our approved ethics protocol includes a risk protocol and risk monitoring by a Safety and Risk Committee. We have added detail to the protocol manuscript about this (p16):

> **"Any adverse events relating to PFA-S training, PFA-S use, or research participation will be reported to the data safety and monitoring committee who will be responsible for stopping the research to prevent harm from occurring."**

We have also added details about the committee, in line with SPIRIT 28a (p30):

> **"Oversight of trial conduct, participant safety, and data integrity will be maintained by an internal data safety and monitoring committee comprising the lead researcher, clinical psychologists, the data manager, and staff liaison. The committee will review adverse events, participant safety issues, trial conduct, and data integrity and report to the wider project guidance research team at regular meetings. Committee members are independent of the study funder and the funder will have no role in monitoring data or trial conduct. A formal committee charter has not been developed due to the low-risk nature of the intervention."**

12. SPIRIT Item 5: no separately accessible Statistical Analysis Plan is provided. Given the large number of primary, secondary, exploratory, predictor/moderator, and implementation outcomes (Table 1 lists well over 20 measures across two respondent groups and four time points), the absence of a separately timestamped SAP is a meaningful gap.

The study includes one primary outcome and one secondary outcome, with remaining outcomes being exploratory or moderator outcomes. The Data Analysis Plan for the single primary outcome and single secondary outcome is provided on p29. We have made some edits to clarify when we are referring to primary and secondary outcomes vs exploratory outcomes.

13. Item 9b analogue: no rationale given for comparator choice. The self-completed checklist marks Item 9b ("explanation for choice of comparator") as "na." A wait-list control is a defensible and common choice for a low-risk school intervention, but SPIRIT explicitly requires this be justified, not marked not-applicable — the rationale (ethical equipoise, eventual access, feasibility) should be stated rather than assumed.

Thank you for pointing out this omission. We have added the following text to the protocol to justify the use of a wait-list control (p9), and updated Item 9b accordingly:

> **"The waitlist framework is an ethical approach that guarantees staff access to the intervention, provides more data (larger N) regarding implementation outcomes, whilst also incentivising participation and minimising attrition."**
